# National Trends in the Adoption of Laparoscopic Cholecystectomy over 7 Years in the United States and Impact of Laparoscopic Approaches Stratified by Age

**DOI:** 10.1155/2014/635461

**Published:** 2014-03-20

**Authors:** Anahita Dua, Abdul Aziz, Sapan S. Desai, Jason McMaster, SreyRam Kuy

**Affiliations:** ^1^Department of Surgery, Medical College of Wisconsin, Milwaukee, WI 53226, USA; ^2^Department of Surgery, Center for Translational Injury Research, University of Texas-Houston, Houston, TX 77030, USA; ^3^Department of Surgery, Queens Medical Centre, Nottingham NG7 2UH, UK; ^4^Department of Surgery, Duke University, Durham, NC 27710, USA

## Abstract

*Introduction*. The aim of this study was to characterize national trends in adoption of laparoscopic cholecystectomy and determine differences in outcome based on type of surgery and patient age. * Methods*. Retrospective cross-sectional study of patients undergoing cholecystectomy. Trends in open versus laparoscopic cholecystectomy by age group and year were analyzed. Differences in outcomes including in-hospital mortality, complications, discharge disposition, length of stay (LOS), and cost are examined. * Results*. Between 1999 and 2006, 358,091 patients underwent cholecystectomy. In 1999, patients aged ≥80 years had the lowest rates of laparoscopic cholecystectomy, followed by those aged 65–79, 64–50, and 49–18 years (59.7%, 65.3%, 73.2%, and 83.5%, resp., *P* < 0.05). Laparoscopic cholecystectomy was associated with improved clinical and economic outcomes across all age groups. Over the study period, there was a gradual increase in laparoscopic cholecystectomy performed among all age groups during each year, though elderly patients continued to lag significantly behind their younger counterparts in rates of laparoscopic cholecystectomy. * Conclusion*. This is the largest study to report trends in adoption of laparoscopic cholecystectomy in the US in patients stratified by age. Elderly patients are more likely to undergo open cholecystectomy. Laparoscopic cholecystectomy is associated with improved clinical outcomes.

## 1. Introduction

It is well established that open cholecystectomy has worse outcomes than laparoscopic cholecystectomy [1–3]. In 1993, the National Institutes of Health (NIH) Consensus Conference on gallstones and laparoscopic cholecystectomy reported lower mortality, decreased disability, shorter LOS, and less patient discomfort with laparoscopic cholecystectomy in the general population and recommended laparoscopic cholecystectomy as the preferred surgical approach [2, 3]. It has been previously demonstrated that elderly patients are more likely to have more complex biliary disease and nearly six times greater odds of mortality following cholecystectomy than their younger counterparts [1–4]. As the proportion of population >65 years old is predicted to rise from 12% to 20% [1] over the next several decades, gallstone disease among the elderly will represent a major surgical burden. However, few studies have examined differences in rates of adoption of laparoscopic cholecystectomy among elderly patients compared with their younger counterparts.

Few studies have examined differences in the adoption of laparoscopic cholecystectomy among elderly patients compared with their younger counterparts. The objective of this study was to characterize national trends in adoption of laparoscopic cholecystectomy performed in the United States (US) and determine differences in outcomes based on laparoscopic or open type procedures by age group.

## 2. Methods

This was a retrospective cross-sectional analysis of inpatient hospitalizations from 1999 to 2006, utilizing the Health Care Utilization Project-Nationwide Inpatient Sample (HCUP-NIS) database, which is a stratified 20% sample of all inpatient admissions to nonfederal, acute care hospitals maintained by the Agency for Healthcare Research and Quality (AHRQ). This is the largest all-payer inpatient database in the U.S., with records from approximately eight million hospital stays each year. Records were limited to adults aged 18–100 years old hospitalized with a diagnosis of cholecystitis, as identified by ICD-9 codes and Clinical Classifications Software (CCS). ICD-9 procedure codes were used to identify all patients who underwent cholecystectomy as the primary procedure during hospitalization (open versus laparoscopic). Patients were stratified into four age groups, aged 18–49, 50–64, 65–79, and ≥80 years. Patient comorbidity was calculated using ICD-9 codes and the Charlson Comorbidity Index.

### 2.1. Independent Variables

Type of procedure (open versus laparoscopic cholecystectomy) was the primary independent variable of interest. For this study, only those patients who carried a diagnosis of cholecystitis and had cholecystectomy as the primary procedure performed during that hospitalization were included.

### 2.2. Outcome Variables

The primary outcome examined was rate of laparoscopic cholecystectomy by year and age group. Outcomes by type of cholecystectomy (open versus laparoscopic) examined were mortality; in-hospital complications; discharge disposition (routine discharge to home versus discharge with home health care or discharge to a short-term hospital, intermediate care facility, or skilled nursing facility), mean length of stay (LOS); and mean total inpatient hospital costs.

Surgical complications were identified using ICD-9 codes and categorized as cardiac, postoperative shock, gastrointestinal, hematologic, renal, pulmonary, infection, thrombosis or embolism, and bile duct injury or repair. Surgical complications were treated as dichotomous variables (none versus ≥1). Mean total inpatient hospital costs were calculated using the HCUP-NIS hospital-specific cost-to-charge ratios (available for 2001–2006) and were standardized to 2006 dollars utilizing the Bureau of Labor Statistics Medical Consumer Price Index [5]. The NIS charge information represents the amount that hospitals were billed for services but does not reflect the actual amount hospital services cost or the specific amounts that hospitals received in payment. In order to see how much to calculate how the hospital charges translate into actual costs, the NIS Cost-to-Charge Ratio Files in the database enable this conversion. Each file contains hospital-specific cost-to-charge ratios based on all-payer inpatient cost for each hospital in the corresponding NIS databases. For this study, cost information was obtained by the NIS database from the hospital accounting reports collected by the Centers for Medicare and Medicaid Services and merged with the appropriate file to the corresponding NIS databases by the data element hospital identification number. Using the merged data elements from the cost-to-charge ratio files and the total charges reported in the NIS databases, the hospital total charge data was converted to cost estimates by multiplying total charges with the appropriate cost-to-charge ratio. Then, using the US Bureau of Labor Statistics Medical Consumer Price Index, they were standardized to 2006 dollars [5].

### 2.3. Statistical Analysis

Bivariate analysis of the independent variables by outcomes was performed using *χ* ^2^ tests for categorical variables and analysis of variance (ANOVA) for continuous variables. Data analysis and management were performed using SAS version 9.1 (Cary, NC, USA). Statistical significance was set at a probability value of *P* ≤ 0.05.

## 3. Results

**Figure 1:**
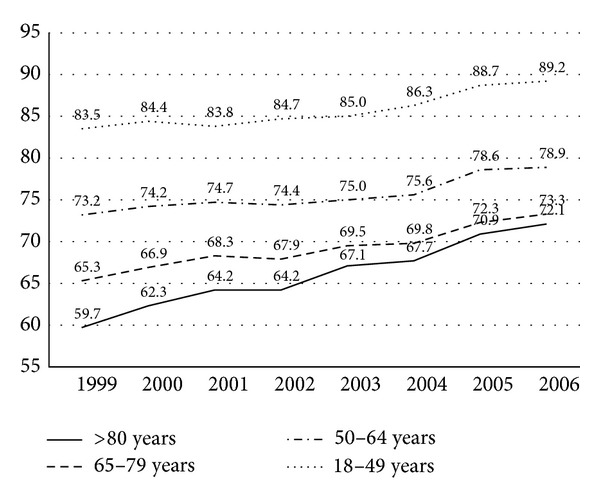
Yearly trends in adoption of laparoscopic cholecystectomy by age group (1999–2006).

Of the 1,332,195 adult admissions for biliary disease in the HCUP-NIS database between 1999 and 2006, cholecystectomy was performed as the primary procedure during hospitalization in 145,675 patients aged 50–64 years, 149,855 patients aged 65–79 years, and 62,561 patients aged ≥80 years. In 1999, representing the start of the study period, laparoscopic cholecystectomy was performed most frequently in patients aged between 18 and 49 years (83.5%) (Figure 1).

In contrast, laparoscopic cholecystectomy was performed less often as the age groups ascended: 50–64 years (73.2%), 65–79 years (65.3%) and >80 years (59.7%). During each year, there was a gradual increase in patients undergoing laparoscopic surgery across all age groups. By the end of the study period in 2006, 89.2% of patients aged 18–49 underwent laparoscopic cholecystectomy. There was also an increase in frequency for patients aged 50–64 years (78.9%) and 65–79 years (73.3%). Interestingly, patients aged >80 years witnessed a marked increase from 59.7% to 72.1%. This represented the biggest increase, of 12.4%, compared to all other age groups.

### 3.1. Outcomes

The mortality rate in the study population undergoing open cholecystectomy increased significantly with advancing age (0.5%, 1.6%, 4.0%, and 8.3%; *P* < 0.001) (Table 1).

However, mortality rates were lower in all age groups with laparoscopic cholecystectomy (0.1%, 0.3%, 0.9%, and 2.3%; *P* < 0.001). Patients aged ≥80 years experienced a significant reduction in mortality following laparoscopic cholecystectomy (open = 8.3% versus laparoscopic = 2.3%; *P* < 0.001) when compared to an open procedure. There was a lower overall rate of surgical complications when comparing laparoscopic to open cholecystectomy between age groups. This was most evident for patients aged 65–79 years (open = 50% versus laparoscopic = 26.3%; *P* < 0.001) and ≥80 years (open = 61.1% versus laparoscopic = 37.2%; *P* < 0.001). The trend in adoption of laparoscopic cholecystectomy was also associated with lower LOS (mean days) when compared to open cholecystectomy among all age groups. In particular, patients aged 65–79 years had significantly shorter LOS (open = 10.29 days versus laparoscopic = 5.01 days; *P* < 0.001) resulting in lower hospital costs (open = $24, 060 versus laparoscopic = $12, 451;  *P* < 0.001). Improved outcomes were also observed in patients ≥80 years with respect to LOS (open = 11.91 versus laparoscopic = 6.77; *P* < 0.001) and hospital costs (open = $26, 342 versus laparoscopic = $15, 030;  *P* < 0.001). In addition, among patients aged ≥80 years, there was a significant increase in these patients being successfully discharged home directly from the hospital (open = 37.0% versus laparoscopic = 63.2%; *P* < 0.001).

## 4. Discussion

Gallstone disease is the most common indication for abdominal surgery in the United States. Prevalence of gallstone disease is known to increase with age. An increasing life expectancy means that, over the next several decades, the proportion of the population aged over 65 years is expected to rise from 12% to 20% resulting in more patients presenting with gallstone disease requiring surgery [1]. Evaluating national trends to decipher the optimum surgical approach for these patients is important.

This large cross-sectional study of hospitalized patients undergoing cholecystectomy found a steady increase in the adoption of laparoscopic cholecystectomy from 1999 to 2006 among all age groups. Although older patients clearly benefited from laparoscopic cholecystectomy than open cholecystectomy, they are significantly less likely than younger patients to undergo laparoscopic cholecystectomy. However, there was an increasing trend among elderly patients (65–79 years and ≥80 years) undergoing toward laparoscopic cholecystectomy over the study period. Compared to younger patients, elderly patients experienced more adverse outcomes overall, but this was reduced with the adoption of laparoscopic surgery. This is important given that elderly patients tend to present with more complex biliary disease and multiple diagnoses that may increase their surgical risk [3]. Elderly patients are also more likely to require further procedures such as cholecystostomy decompression, endoscopic retrograde cholangiopancreatography (ERCP), common bile duct exploration, and intraoperative cholangiogram [3, 4]. Among the elderly, laparoscopic cholecystectomy, compared with open cholecystectomy, is associated with lower mortality rates, surgical complications, LOS, hospital costs, and better discharge deposition overall, supporting laparoscopic cholecystectomy as the procedure of choice in this population.

In previous studies, the mortality rates in elderly patients undergoing laparoscopic cholecystectomy are reported between 0 and 1% [6–8]. However, these studies defined elderly as patients between the ages of 65 to 79 years only. In direct comparison, we observed a comparable mortality rate of 0.9% in the same age group. In our study cohort of patients aged ≥80 years, the mortality rate was 2.3% among those undergoing laparoscopy compared to 8.3% among those undergoing an open procedure.

This study also observed lower rates in morbidity among elderly patients undergoing laparoscopic compared with open cholecystectomy. Compared to younger patients, the rates were higher in the elderly population overall. However, rates significantly declined when comparing open versus laparoscopic cholecystectomy in the aged-matched elderly groups. Clinically, this translated to reduced LOS and decreased disability, reflected by the fact that more patients were primarily discharged home directly from hospital. Legner et al. [9] reported that elderly patients discharged to an institutional care facility following abdominopelvic surgery had a 4-fold increase in 1-year mortality [9]. Based on this finding, it would appear that elderly patients undergoing laparoscopic compared with open cholecystectomy may also have improved 1-year outcomes given that higher numbers of these patients were discharged directly to their homes.

To our knowledge, this is the largest, comprehensive study of its kind examining the trends in adoption of laparoscopic cholecystectomy among elderly patients and outcomes of laparoscopic cholecystectomy in this population. Other studies examining outcomes of laparoscopic cholecystectomy in the elderly population have been limited to single institutions [10–16]. The findings from such studies are comparable to our study describing decreased morbidity and mortality, LOS, and surgical complications among elderly patients undergoing laparoscopic cholecystectomy. Our study validates these single institutional findings using a large generalizable sample from across the US. While current literature supports the role of laparoscopic cholecystectomy in elderly patients, it should also be acknowledged that elderly patients more commonly present with complex biliary disease resulting in increased complication rates and mortality regardless of procedure choice (open or laparoscopic).

*Limitations.* This was a retrospective study of a large database and hence limitations inherent to any administrative database such as HCUP-NIS are noted. A limitation of this study was the fact that there is no ICD-9 code for conversion from laparoscopic to open cholecystectomy; hence, we were unable to calculate the rate of conversion. However, HCUP-NIS is a well-validated and rigorously maintained database with a low error rate. Operative details such as operative duration, findings of gangrenous or perforated cholecystitis, postdischarge followup including readmissions, and postdischarge mortality are not available in HCUP-NIS. A limitation of the NIS database, however, is that ASA class is not available. We were able to assess the comorbidities of the elderly and the nonelderly patients, but the focus of this study is on the discrepancy in the rates of performance of laparoscopy in the elderly compared with the nonelderly. While we could not match the elderly and the nonelderly by comorbidity or severity of biliary 12 disease, we did note in this epidemiological study that the performance of laparoscopic cholecystectomy in the elderly group was lagging compared to their younger counterparts. The comparative effectiveness of laparoscopic over open cholecystectomy is clear from previous data with the advantage of laparoscopy.

This epidemiological study delineates that the performance of a laparoscopic approach for cholecystectomy in the elderly lags behind their younger counterparts.

## 5. Conclusion

In this large nationwide cross-sectional study of patients undergoing cholecystectomy, we observed an improvement in clinical outcomes for all patients in the laparoscopic arm with a large benefit noted in elderly patients. This coincided with an increasing trend in the adoption of laparoscopic cholecystectomy. Though elderly patients experienced a significant benefit in laparoscopic surgery, with fewer postoperative complications and lower mortality rates, they still lag significantly behind younger patients in undergoing laparoscopic cholecystectomy. However, we recognise that more data is needed, including data of elderly patients managed as outpatients and the investigation of 1-year mortality rates. Laparoscopic cholecystectomy is a valid primary option for biliary disease and should be considered the procedure of choice in all age groups.

## Figures and Tables

**Table 1:** Outcomes by type of cholecystectomy procedure (laparoscopic versus open).

Patient outcomes	≥80 years old			65–79 years old			45–64 years old			18–44 years old		
	Laparoscopic (%)	Open (%)	*P* value	Laparoscopic (%)	Open (%)	*P* value	Laparoscopic (%)	Open (%)	*P* value	Laparoscopic (%)	Open (%)	*P* value
Surgical complications (%)	37.20	61.10	<0.0001	26.30	50.00	<0.0001	16.40	37.20	<0.0001	10.30	25.30	<0.0001
Mortality (%)	2.30	8.30	<0.0001	0.90	4.00	<0.0001	0.30	1.60	<0.0001	0.10	0.50	<0.0001
LOS (mean days)	6.77 (SD 6.46)	11.91 (SD 10.07)	<0.0001	5.01 (SD 5.72)	10.29 (SD 10.34)	<0.0001	3.8 (SD 4.8)	8.1 (SD 9.6)	<0.0001	3.1 (SD 3.4)	6.2 (SD 7.9)	<0.0001
Cost (mean in 2005$)	15,030 (SD 15,100)	26,342 (SD 27,611)	<0.0001	12,451 (SD 13,723)	24,060 (SD 30,200)	<0.0001	10,425 (SD 11,369)	19,651 (SD 27,588)	<0.0001	8,858 (SD 8,051)	15,723 (SD 21,811)	<0.0001
Discharge disposition (%)			<0.0001			<0.0001			<0.0001			<0.0001
Routine home	63.20	37.00		86.60	66.00		95.80	85.30		98.40	92.70	
Short-term hospital	1.10	1.20		0.70	1.10		0.50	0.80		0.40	0.70	
Intermediate care	21.80	37.30		6.20	14.60		1.50	4.30		0.50	1.70	
Home health	11.60	16.20		5.60	14.30		1.80	7.80		0.60	4.20	
Against medical advice	0.07	0.04		0.07	0.06		0.12	0.13		0.13	0.20	
Died	2.30	8.27		0.87	3.95		0.26	1.61		0.06	0.53	
Unknown	0.01	0.04		0.01	0.01		0.01	0.01		0.00	0.01	
